# Bio-Guided Isolation of Prospective Bioactive Constituents from Roots of *Clausena indica* (Dalzell) Oliv

**DOI:** 10.3390/molecules24244442

**Published:** 2019-12-04

**Authors:** Nguyen Van Quan, Tran Dang Xuan, La Hoang Anh, Hoang-Dung Tran

**Affiliations:** 1 Department of Development Technology, Graduate School for International Development and Cooperation, Hiroshima University, Hiroshima 739-8529, Japan; nguyenquan26@gmail.com (N.V.Q.); hoanganh6920@gmail.com (L.H.A.); 2 Faculty of Biotechnology, Nguyen Tat Thanh University, 298A-300A Nguyen Tat Thanh Street, Ward 13, District 4, Ho Chi Minh City 72820, Vietnam; thdung@ntt.edu.vn

**Keywords:** *Clausena indica*, dentatin, nordentatin, clausine K, antioxidants, anti-α-amylase, anti-elastase, anti-tyrosinase

## Abstract

*Clausena indica* fruits are routinely used for the culinary purpose as natural spices, whereas leaves and roots are folk medicine with various health benefits in southern China, South and Southeast Asia. In this study, the bioassay-guided fractionation by column chromatography yielded three pure compounds including dentatin, nordentatin, and clausine K and five active fractions (**Re1-5**) from *C. indica* roots. These known anticancer compounds were confirmed by X-ray diffraction, ^1^H-, ^13^C-nuclear magnetic resonance (NMR), and electrospray ionization tandem mass spectrometric (ESI-MS-MS) analyses. Meanwhile, the phytochemical constituents from fractions were identified by gas chromatography-mass spectrometry (GC-MS). The isolates, fractions’ components and their biological activities were first time investigated on *C. indica*. By in vitro DPPH and ABTS scavenging assays, nordentatin (IC_50_ = 49.2 and 69.9 µg/mL, respectively) and the fraction **Re4** (32.4 and 38.5 µg/mL, respectively) showed the strongest antiradical activities, whereas clausine K presented a moderate and dentatin had negligible antioxidant activity, respectively. The anti-α-amylase activity of *C. indica* root extracts was mainly attributed to the fraction **Re2** which inactivated the enzymatic assay with IC_50_ of 573.8 µg/mL. Among tested samples, only nordentatin and clausine K were effective in the pancreatic elastase inhibition, however, their influences were trivial. Markedly, clausine K and **Re4** performed the most remarkable tyrosinase inhibition with IC_50_ values of 179.5 and 243.8 µg/mL, respectively, which were in turn 4 and 3 times stronger than myricetin (IC_50_ = 735.6 µg/mL), a well-known tyrosinase inhibitor. This is the first report affirming clausine K to be a new strong tyrosinase inhibitor. Isolated compounds from *C. indica* roots were quantified by high-performance liquid chromatography (HPLC), of which, dentatin, nordentatin, and clausine K accounted for 14.74, 6.14, and 1.28 mg/g dry weight. The findings suggest that bioactive constituents from *C. indica* roots may be potentially employed for the development of antidiabetic, antiaging and cosmetic agents.

## 1. Introduction

*Clausena indica* (Dalzell) Oliv. is an evergreen small tree that belongs to the citrus family, Rutaceae. The plant is widely distributed in South China and South and Southeast Asia [1]. The *C. indica* species is traditionally used for multiple purposes as foodstuffs and folk medicines. In Vietnam, leaves and fruits of *C. indica* are mainly used for cooking as natural spices, which can increase the food’s flavor and attractivity. Besides, its leaves and roots are customarily utilized in curing a cold, flu, headache, colic, and rheumatism. The leaf essential oil possesses antimicrobial and antibacterial activities [1,2]. Numerous phytochemical components were earlier reported in *C. indica* roots by Joshi and coworkers, of which coumarins, carbazole alkaloid (6-methoxyheptaphylline), and sesquiterpenes were the lead compounds [3,4,5,6]. On the other hand, plants in the *Clausena* genus were scientifically documented as a diverse source of anticancer agents, in which coumarins and carbazole alkaloids are the most dominant anti-carcinogenic compounds isolated from this genus [7]. While carbazole alkaloids have been found from various *Clausena* species, coumarins have been detected mostly in *C. harmandiana* and *C. excavata* [7]. However, the compounds from *C. indica* roots and their involvement in the biological activities of this species have not been investigated adequately.

In humans, reactive oxygen species (ROS) are invariably produced in the body via redox reaction [8]. ROS are well known as potential products having harmful effects on the essential constituents of living cells [9] which may result in a serious development of oxidative stress and many human diseases such as skin pigmentation, type 2-diabetes, and cancers [10]. Recently, a strong association among skin manifestations, diabetes and obesity were reported with trustworthy documentation in both clinical and practical trials [11,12]. Therefore, studies on antioxidants can be regarded as the prerequisite for various related anti-disease effects such as diabetes and skin diseases. In the context of the current drug development, antioxidants that have natural provenance are the most selective priority owing to their advantageous safety and availability [13]. Some outstanding sources such as phenolics, flavonoids, and secondary metabolites, especially derived from plants, are being widely exploited in the pharmaceutical industry [14]. The phytochemicals with health benefits are expected to be considered as a substitute for synthetic drugs that may cause various side-effects. Hence, the discovery of natural metabolites exhibiting a remarkable resistance to a wide range of dangerous diseases can be a potential approach in phytochemicals and pharmacological studies.

The above-mentioned rationales prompted us to conduct this study aiming to isolate bioactive compounds from the extract of *Clausena indica* roots and to examine their contributions to antioxidant, antidiabetic, and anti-skin-aging properties. This study proposes effective analytical methods to isolate and purify bioactive constituents from *C. indica* and give insights to future research targeting to novel drugs that can control multiple diseases.

## 2. Results and Discussion

### 2.1. Isolation, Identification, and Confirmation of Bioactive Compounds from Clausena Indica Roots

**Figure 1:**
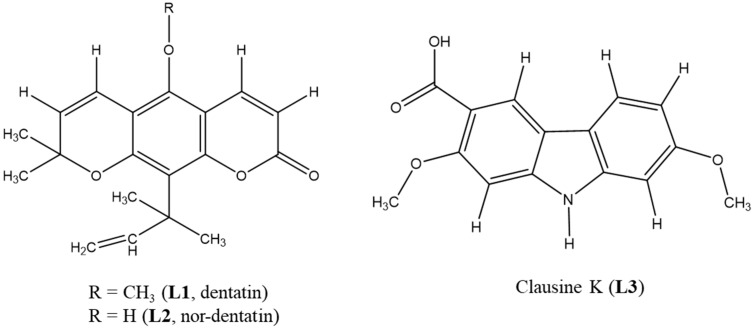
Chemical structures of isolated compounds from *Clausena indica* roots.

The ethyl acetate extract (MRE, 7g) separated from total methanolic extract (MRT) accounted for 4.6% of the dry weight of the sample. By a normal phase column chromatography, MRE was fractionated into five fractions **Re1**–**Re5** which were 1.4%, 0.4%, 0.7%, 0.1%, and 0.3% of the extraction efficiency, respectively. Meanwhile, the yields of isolated compounds dentatin (**L1)**, nordentatin (**L2)**, and clausine K (**L3**) were 0.5%, 0.4%, and 0.1%, respectively. The three compounds were isolated from *C. indica* roots for the first time (Figure 1).

Chemical structures of purified metabolites refined by X-rays diffraction (Table 1), 1D-^1^H- and ^13^C-nuclear magnetic resonance (NMR) and electrospray ionization mass spectrometry (ESI-MS) were identical compared with those in literature [15,16] (Supplementary data, Tables S1–S3, Figures S1–S3).

Along with three purified metabolites, phytochemical contents of *C. indica* roots were preliminarily identified by gas chromatography-mass spectrometry (GC-MS). The results are delineated in Table 2. Among 18 compounds detected from two extracts (MRT and MRE) and five fractions (**Re1**–**Re5**), 10 compounds were certainly confirmed including palmitic acid [17], seselin [18], l-5-propylthiomethylhydantoin [19], braylin [20], anisocoumarin B [21], dentatin [16], nordentatin [16], clausine K [16], 7-hydroxyheptaphylline [16], clionasterol [22]. On the other hand, three unknown compounds 1 (**UC1**), 2 (**UC2**) and 3 (**UC3**) were found at retention times 20.24, 20.85 and 21.08 min, respectively. Of which, **UC1** was the most abundant compound in **Re3** (58.7%) and **Re4** (26.1%) while **UC3** was only observed in Re3 (2.8%). **UC2** was 2.1% and 2.3% in **Re3** and **Re5**, respectively. The mass spectra of **UC1-3** by GC-MS are supplied in Supplementary data, Figure S4. Additionally, five compounds were tentatively identified as carbazole alkaloids (**CA1-5**) by comparing their ion-fragment composition and molecular weight with those in literature data. Accordingly, **CA1** (RT 21.85 min, 227 g/mol) might be either clausine O, V [15,23] or clauszoline N [24]. **CA3** (RT 23.34, 241 g/mol) was probably clausines E, I, M, P [25,26] or 1-hydroxy-7-methoxy-9*H*-carbazole-3-carbaldehyde [27]. **CA4** (RT 24.85, 279 g/mol) could be clauraila B [15], or heptaphylline [23], or clausine D [28]. Meanwhile, **CA2** (RT 23.29, 293 g/mol) and **CA5** (RT 24.92, 285 g/mol) might be clauraila D [15] and clausine H [26], respectively. Theoretically, **UC2** could be recognized as either suberosin or tryptamine isovalerate but its mass spectrum showed three precursor peaks at *m/z* 229, 201, and 244 which were apparently peculiar (Figure S4). Also, the mentioned compounds were previously reported in *C. excavata*. Therefore, advanced spectroscopic techniques and isolating pure compounds should be further applied and carried out in order to increase the reliability of compound identifications from *C. indica*.

The phytochemical components from the roots of *C. indica* were firstly reported by Joshi and Gawad in 1971 [3], in which, five furanocoumarins including imperatorin, phellopterin, chalepensin, and chalepin were considered as the first compounds isolated from *C. indica* roots. Later studies have isolated various coumarins, amides and carbazole alkaloids from this plant [3,4,5,6,29,30]. Apart from those components, terpenes and volatile constituents were investigated with potential antimicrobial and antibacterial activities in the essential oil of *C. indica* aerial parts [1,2,31]. In the present study, the first detection of dentatin, nordentatin, clausine K and other promising alkaloids may contribute to the diversity of natural products as well as the medicinal potentials of *C. indica*.

### 2.2. Biological Activities of Clausena Indica

The antioxidant capacities of *C. indica* extracts, fractions, and purified compounds were evaluated via 2,2-diphenyl-1-picrylhydrazyl (DPPH) and 2,2′-azino-bis (ABTS) radical scavenging tests and lipid peroxidation inhibitory assay (β-carotene bleaching inhibition), see Table 3. Noticeably, the fraction **Re4** was the strongest radical scavenger, which showed IC_50_ values of 32.4 and 38.5 µg/mL at DPPH and ABTS assays, respectively. Among isolated compounds, nordentatin exhibited the highest antioxidant ability (IC_50_ = 49.2 and 69.9 µg/mL for DPPH and ABTS assays, respectively) followed by clausine K with IC_50_ values of 2197.8 and 5264.0 µg/mL, respectively. Both nordentatin and **Re4** performed higher antioxidant activity than BHT (IC_50_ = 82.8 µg/mL) in the ABTS assay. In contrast, dentatin had negligible effects in both radical scavenging assays. In terms of β-carotene bleaching inhibition assay, MRE (82.8%) extract and **Re5** (81.2%) presented strongest lipid peroxidation inhibitions (LPI) which were in line with BHT (82.7%). Among purified compounds, the LPI of nordentatin (69.8%) was significantly higher than that of clausine K (60.6%), whereas dentatin had an inconsequential activity (37.9%). These results were coincident with previous reports [16,32], by which nordentatin was proven as a potent antioxidant whereas dentatin was a weak or inactive radical scavenger. On the contrary, studies on the antioxidant activity of clausine K are limited. Songsiang and coworkers reported that clausine K from *C. harmandiana* was inactive in the DPPH assay and a trivial LPI by malondialdehyde assay [16]. In the present research, by using different modified in vitro models, we agree that clausine K from *C. indica* is an antioxidant though its activity is not potent. Considerably, at a concentration of 500 µg/mL, clausine K indicated 60.6% of LPI by the β-carotene bleaching assay. The obtained results implied that the DPPH assay used acetate buffer (pH 5.5) and β-carotene bleaching might be the proper approach to figure out the antioxidant activity of clausine K. Also, previous study confirmed the acid buffer could contribute a stimulatory effect on DPPH radical scavenging speed [33]. However, in vivo assays should be further conducted to properly evaluate the antioxidant potential of clausine K and its applicability. Furthermore, the forceful antioxidant property of **Re2-4** might be attributed to the synergic activity of **UC1-3**, anisocumarin B, nordentatin, **CA1**, **CA3-5**. Remarkably, **UC2** and **CA4** could be the most significant contributors because these two metabolites together with nordentatin were mostly detected in the active fractions **Re2-4**.

By in vitro enzymatic assays, crude extracts, fractions, and isolated compounds of *C. indica* showed various levels of inhibitory actions on key enzymes related to diabetes (α-amylase) and skin aging (elastase and tyrosinase) (Table 4). Particularly, *C. indica* had a slight inhibitory effect towards α-amylase reaction, in which, the inhibition was displayed by crude extracts MRT (IC_50_ = 1383.3 µg/mL), MRE (IC_50_ = 1004.6 µg/mL) and the fraction **Re2** (IC_50_ = 573.8 µg/mL). The results might be explained by the exclusive existence of palmitic acid, clionasterol and **CA3** in the fraction **Re2** (Table 2). Previous studies also demonstrated the effective anti-diabetic activity of palmitic acid [34] and clionasterol [35]. However, the tentative carbazole **CA3** might play a role in the total inhibitory activity of *C. indica* root extracts.

Among tested samples, only nordentatin and clausine K were found to inhibit pancreatic elastase though their activity was not strong. At the maximum concentration of 10 mg/mL, nordentatin and clausine K suppressed 41.9% and 21.9% of the elastase action. Markedly, fractions **Re3**, **Re4**, **Re5**, and clausine K flaunted considerable inhibition on tyrosinase (Table 4). Clausine K (IC_50_ = 179.5 µg/mL) and **Re4** (IC_50_ = 243.8 µg/mL) were the strongest tyrosinase inhibitors, which were in turn 4 and 3 times stronger than myricetin (IC_50_ = 735.6 µg/mL), a well-known tyrosinase inhibitor. This is the first report of clausine K as a new promising tyrosinase inhibitor. Previous studies indicate elastase and tyrosinase are two key enzymes related to the degradation of skin’s elasticity and the formation of skin’s freckles due to the excessive UV exposure and ROS accumulation in the body [36,37]. Inhibitions of elastase and tyrosinase can prevent both skin wrinkles and skin browning. Therefore, the discovery of potential natural tyrosinase inhibitors from *C. indica* root extracts might be a good suggestion for the cosmetic industry.

Basically, the biological activity of a mixture is not only defined by the content of compounds but also relies upon the compound type or its chemical structure, in which, the functional group plays a crucial role. While the antioxidant activity is merely characterized by the number of hydrogen donors, the enzymatic inhibitory activity is more complicated and is defined by various factors of the chemical structure aspect [38]. For instance, an anti-α-amylase flavonoid requires to possess hydrogen bonds between the hydroxyl groups of the polyphenol ligands and the catalytic residues of the binding site [39]. In the case of mimosine, a known tyrosinase inhibitor from Mimosoideae family, its structure contains an active pyridine ring linked with the ketohydroxy and amino carboxylic groups which may exert its potent biological activities [40]. To be more specific, the position of functional groups along with bonding types are considered as the prerequisite determinants for the bioactivity of a compound. In the present study, the replacement of the hydroxy group by the methoxy one at C-5 of the seselin skeleton resulted in an inactive antioxidant and elastase inhibitor, such as dentatin. Furthermore, the results suggested that the carbazole-like structure is more inhibitory of tyrosinase reaction rather than the seselin coumarin structure. However, dentatin, nordentatin and clausine K were manifested to be antibacterial [41], antifungal [42], antiplasmodial [19], anti-HIV-1 [43,44], and anti-hepatitis B virus (HBV) [45] agents. Moreover, these compounds were substantiated with persuasive anticarcinogenic properties in various cancer cells including colon, lung, oral human epidermal carcinoma, kidney, cholangiocarcinoma and others [7,15,16,32,46]. Hence, the quantification of these precious phytochemicals is genuinely essential for researching and developing natural drugs.

### 2.3. Dentatin, Nordentatin and Clausine K Contents in Clausena indica Roots’ Extract

**Figure 2:**
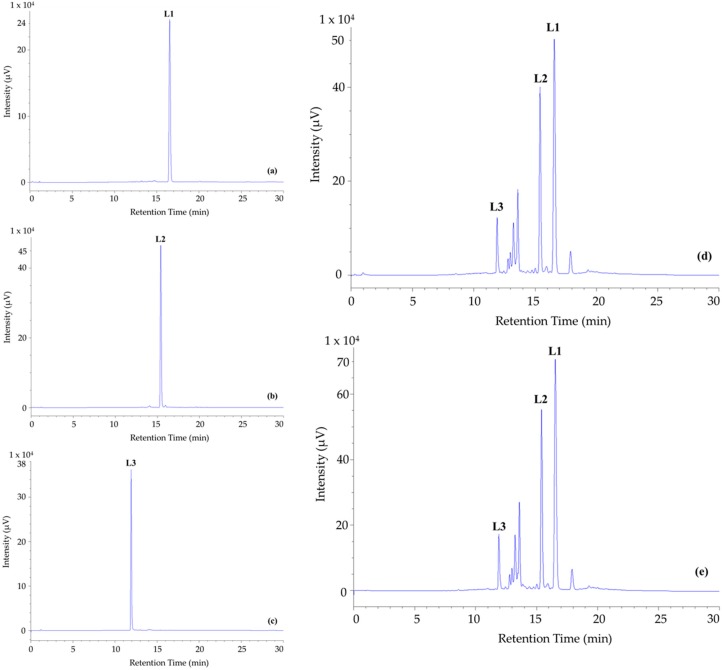
High-performance liquid chromatography (HPLC) chromatographic profile at 270 nm of isolated compounds from *Clausena indica* roots. (**a**) dentatin-**L1**; (**b**) nordentatin-**L2**; (**c**) clausine K-**L3;** (**d**) methanolic extract (MRT); (**e**) ethyl acetate extract (MRE).

The contents of dentatin, nordentatin and clausine K were successfully quantified by using high-performance liquid chromatography (HPLC). The HPLC chromatogram of MRT and MRE extracts and purified compounds from *C. indica* root is sketched in Figure 2. A good separation of phytochemicals under 270 nm was observed, in which, dentatin, nordentatin and clausine K were detected at the retention times of 16.5, 15.4, and 11.9 min, respectively. The similar components obtained from HPLC for MRT (Figure 2d) and MRE (Figure 2e) extracts suggested the ethyl acetate separation could gain most of the major compounds from the *C. indica* root. Basing on the linear equation established from the calibration curve, the quantities of dentatin, nordentatin, and clausine K in the MRT extract were correspondingly measured as 14.74, 6.14, and 1.28 mg/g dry weight of *C. indica* roots.

In fact, the quantification of the bioactive substances dentatin, nordentatin, and clausine K by HPLC-UV were described by Wangboonskul and colleagues [47]. In the present study, we developed a new system by simplifying the gradient component coupled with a reversed-phase HPLC column that helped to increase the sensitivity and shorten the operation time. In particular, in this study, the limits of detection (LOD) and the limits of quantitation (LOQ) values for dentatin, nordentatin, and clausine K were more sensitive than those of Wangboonskul’s method by 625 and 514, 392 and 316, 188 and 253 times, respectively.

The quantified amounts of dentatin, nordentatin, and clausine K by HPLC were in line with those of the isolated compounds by column chromatography (dentatin > nordentatin > clausine K). The HPLC results suggested that other active components in *C. indica* roots could be prospectively detected by using advanced techniques, such as liquid chromatography equipped with tandem mass spectrometry (LC-MS-MS). The use of a reversed-phase column and gradient system described in this study can be employed and incorporated with more sensitive detectors and separators in order to purify and elucidate more bioactive components in *C. indica* roots.

## 3. Materials and Methods

### 3.1. General Experimental Procedures

The X-ray intensity data were measured on a Bruker D8 goniometer system equipped with a Bruker Turbo X-ray Source rotating-anode X-ray tube (MoKa, λ = 0.71073 Å) and a multilayered confocal mirror monochromator. ^1^H- and ^13^C-NMR spectra were acquired on a Bruker Ascend 400 MHz NMR spectrometer (BRUKER BioSpin, Faellanden, Switzerland) at 400 and 125 MHz, respectively. LTQ Orbitrap XL mass spectrometer (Thermo Fisher Scientific, Waltham, MA, USA) equipped with a source of electrospray ionization (ESI) was employed to get ESI-MS spectra of pure compounds. A Jasco HPLC system consisting of PU-4180 RHPLC pump, LC-Net II/ADC controller, and UV-4075 UV/Vis detector (Jasco, Tokyo, Japan) and the GC–MS system (JMS-T100 GCV, JEOL Ltd., Tokyo, Japan) were used to identify bioactive constituents. Biological activities were in vitro assayed by using a Multiskan^TM^ microplate spectrophotometer (Thermo Fisher Scientific, Osaka, Japan) and U-shape microplates (Greiner Bio-one, Monroe, NC, USA). Reagents, solvents, and chemicals at high grades were purchased from Fujifilm Wako Pure Chemical Corporation (Osaka, Japan), Junsei Chemical Co., Ltd. (Tokyo, Japan), Fisher Scientific company (Hampton, NH, USA) and Sigma-Aldrich (St. Louis, MO, USA).

### 3.2. Materials

Roots of *Clausena indica* were collected in Bac Kan province, Vietnam in March 2019. The identification of the species was morphologically done basing on Plants Database Missouri Botanical Garden, United States (TROPICOS—http://www.tropicos.org) and Vietnam Plant Data Center (http://www.botanyvn.com). The voucher specimen number MMR-J2019 was preserved at the Laboratory of Plant Physiology and Biochemistry, IDEC, Hiroshima University, Japan.

### 3.3. Sample Preparation, Extraction and Isolation

The collected *C. indica* roots were washed by tap water then immersed in 0.5% sodium hypochlorite (NaClO) for 30 min to remove soil, bacteria and other impurities. Afterward, the sample was washed several times with distilled water and dried at 40 °C by a convection oven for 10 days. The dried roots were shredded and ground to make powder. Subsequently, the root powder (140 g) was extracted 3 times by 500 mL of methanol (MeOH, 99.8%) for 5 days. After combination, MeOH was evaporated by the Rotavapor^®^ R-300 (Nihon Buchi K.K., Tokyo, Japan) and the total crude extract (MRT, 11 g) was obtained. The MRT was homogenized in 100 mL distilled water, then fractionated by hexane and ethyl acetate to yield hexane (MRH, 82 mg) and ethyl acetate (MRE, 7.2 g), respectively. A screening on biological activities of all extracts and fractions was preliminarily implemented. The strong active extracts were selected for further analysis. The negligible or none active ones (aqueous residue) were discarded.

**Figure 3:**
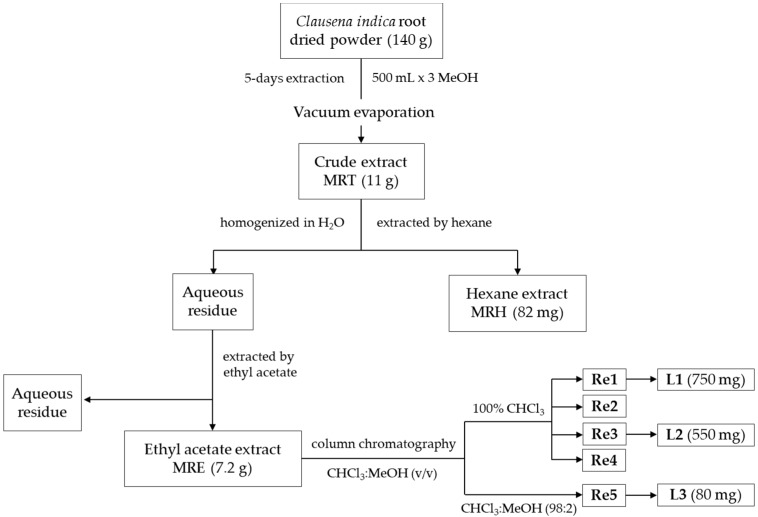
Isolation procedure of bioactive constituents from *Clausena indica* root extract.

The MRE extract was subjected to column chromatography over silica gel (200-400 mesh, 40 g), using chloroform/methanol as mobile phase (100:0 to 98:2, *v*/*v*) to yield 5 fractions (**Re1**–**Re5**). Out of the separated fractions, **Re1**–**Re4** fractions were collected from the 100% chloroform elution, meanwhile, **Re5** was obtained from the chloroform/MeOH (98:2, *v*/*v*) elution. After drying at room temperature, three compounds were purified including **L1** (750 mg) from Re1, **L2** (550 mg) from Re3, and **L3** (80 mg) from Re5. Similarities among fractions and purities of isolated compounds were tested by thin-layer chromatography (TLC) and HPLC. Extracts, fractions and pure compounds were dissolved in MeOH and dimethyl sulfoxide (DMSO) for further biological assays. The extraction and isolation processes are delineated in Figure 3.

### 3.4. Biological Assays

Samples were prepared in either methanol or DMSO with a range of concentrations 0.01–10 mg/mL in bio-assays. Each assay was conducted in triplicate and repeated three times. The dose-response curve for each sample was obtained and used to establish the corresponding linear curve for IC_50_ value calculation, see supplementary data, Figures S5 and S6.

#### 3.4.1. Antioxidant Activities

The antioxidant capacity of bioactive constituents from *C. indica* roots was examined by DPPH radical scavenging, ABTS radical cation discoloration and β-carotene bleaching assays following methods described previously [37] with minor modifications.

In DPPH assay, the mixture of the methanolic sample (80 µL), 0.5 mM DPPH (40 µL) and 80 µL of acetate buffer (0.1 M, pH 5.5) was incubated for 15 min at 25 °C [33]. The absorbance was recorded at 517 nm by the microplate reader. Whereas, the working solution of ABTS radical cation was prepared by the same procedure [37] prior to checking with *C. indica* samples. Briefly, 2.45 mM potassium persulfate was mixed with aqueous ABTS (7 mM) solution (1:1, *v*/*v*) and incubated in darkness at room temperature for 16 h. The working solution was then obtained by diluting ABTS cation solution in methanol. In a well of the microplate, 20 µL of methanolic sample was mixed with 180 µL of the above working solution. The mixture was then incubated for 20 min at 25 °C and the absorbance was read at 734 nm. Both DPPH and ABTS assays were conducted under the avoidance of light. Butylated hydroxytoluene (BHT) was used as positive control while MeOH was negative one. The radical scavenging percentage was calculated as follows:

Radical scavenging activity (%) = [1 − (S − B_S_)]/(C − B_C_) × 100 (1)

where C is the absorbance of the reaction with negative control (methanol), S is the absorbance of reaction with sample or positive control (BHT), B_C_ (blank of control) is the absorbance of negative control sample without radical solution, B_S_ (blank of sample) is the absorbance of tested sample without radical solution. The IC_50_ value was determined as the concentration needed to reduce 50% of the radical absorbance by a linear equation acquired from the dose-response curve (Figures S5 and S6).

In β-carotene bleaching test, 25 µL of methanolic samples (500 µg/mL) was blended with 200 µL of an emulsion containing β-carotene (4 µg/mL) and linoleic acid (0.04%). The reaction was implemented at 45 °C and the reduction of β-carotene was recorded at 492 nm every 15 min up to 180 min. The antioxidant activity was expressed by the lipid peroxidation inhibition (LPI in %) which was calculated as the earlier formula [37].

#### 3.4.2. α-Amylase Inhibitory Assay

Porcine pancreatic α-amylase (PPA) inhibition of *C. indica* extracts and isolated compounds was determined by the starch-iodine method [48]. In summary, a tested sample (20 µL) was incubated with an equal volume of PPA (20 U/mL) at 37 °C for 10 min. The reaction was initiated by adding 30 µL of starch solution (0.5%). The reaction was performed in 8 min at 37 °C before being stopped by 1M HCl and then fixing by 100 µL of 0.25 mM iodine. The suppression of starch digestion by tested samples was measure at 565 nm. The inhibitory activity was delineated by the IC_50_ value. Acarbose was used as a reference.

Inhibition percentage (%) = (A − C)/(B − C) × 100(2)

where A is the absorbance of reaction with the presence of sample or acarbose, B is the absorbance of reaction without enzyme, C is the absorbance of reaction with the absence of sample or acarbose.

#### 3.4.3. Elastase and Tyrosinase Inhibitory Assay

The inhibitory effects of *C. indica* extracts and isolated compounds on the key enzymes related to skin problems elastase and tyrosinase were assessed by the protocol reported by Chompoo et al. [35] with several adjustments.

In particular, 20 μL of a sample (in DMSO) was added to 180 μL of the substrate buffer (1 mM SANA in 0.1 M Tris-HCl buffer, pH 8.0). The solution was mixed and maintained for 5 min at 25 °C. The reaction was started by adding 20 μL of pancreatic elastase (0.1 U/mL). After 12 min of incubation at 25 °C, the absorbance was measured at 410 nm. Oleanolic acid and DMSO were used as positive and negative controls, respectively.

Twenty microliters of sample were mixed with 120 μL of phosphate buffer (20 mM, pH 6.8) and 20 μL of tyrosinase (500 U/mL in buffer), respectively. After a five minutes-incubation at 25 °C, 50 μL of 2 mM L-tyrosine substrate was pipetted to start the reaction. The mixture was slightly shaken and incubated for another 10 min at 25 °C prior to being measured under 470 nm. Myricetin and vanillin were used as positive references whereas DMSO was the negative control. The inhibition percentage of enzymatic assays was computed as:

Inhibition (%) = [1 − (C − D)/(A − B)] × 100(3)

where A is the absorbance of the control with the enzyme, B is the absorbance of the control without the enzyme, C is the absorbance of the sample with the enzyme, and D is the absorbance of the sample without the enzyme. The IC_50_ value was defined as the concentration of a sample that suppressed 50% of the enzymatic reaction over the negative control (Figures S5 and S6).

### 3.5. Identification and Confirmation of Isolated Compounds and other Phytochemical Constituents

The structures and chemical formula of isolated compounds L1, L2 and L3 were identified and confirmed by X-ray diffraction, ^1^H- and ^13^C-NMR and the flow injection analysis (FIA) coupled with electrospray ionization tandem mass spectrometric (FIA-ESI-MS-MS). By X-ray experiment, the crystal sample was exposed to X-ray for 0.80 hours at −100 °C. The frames were integrated with the Bruker SAINT software package using a narrow-frame algorithm. Data were corrected for absorption effects using the Multi-Scan method (SADABS). The structure was solved and refined using the Bruker SHELXTL software package. Meanwhile, ^1^H- and ^13^C-NMR spectra were analyzed and elucidated by Bruker Topspin software version 3.2. Additionally, MS-MS spectra of isolated compounds were acquired by FIA-ESI-MS-MS using a positive model. In summary, isolated compounds were dissolved in MeOH:acetonitrile (1:1, *v*/*v*) to make a concentration of 100 µg/mL. One microliter of each sample was automatically injected to the ESI system which was set up as follows: ion source voltage (4.5 kV), capillary voltage (50 V), tube lens offset (80 V), capillary temperature (330 °C), vaporizer temperature (−65 °C). The gas carrier was nitrogen with the sheath flow rate of 50 arb, and the aux flow rate of 10 arb [49]. The full-scan and data-independent scan spectra for MS-MS on [M + H]^+^ were achieved and processed by using Xcalibur software integrated with the NIST database. The online database (Pubchem, National Center for Biotechnology Information, U.S. National Library of Medicine, Bethesda, MD, USA) and the literature were used for references.

*Dentatin* (**L1**): pale yellow plate (CHCl_3_), ESI-MS *m*/*z* 327.1593 [M + H]^+^ (calcd for C_20_H_23_O_4_, 327.1596), Figure S1, crystal data see Table 1. ^1^H-NMR and ^13^C-NMR signals were closely coincident with those reported in literatures (Table S1) [16,25,32,41].

*Nordentatin* (**L2**): light yellow block (MeOH), ESI-MS *m*/*z* 313.1434 [M + H]^+^ (calcd for C_19_H_21_O_4_, 313.1440), Figure S2, crystal data see Table 1. ^1^H-NMR and ^13^C-NMR data were in good agreement with those in the literature (Table S2) [16,25,32].

*Clausine K* (**L3**): clear yellow block (DMSO), ESI-MS *m*/*z* 272.0914 [M + H]^+^ (calcd for C_15_H_14_O_4_N, 272.0923), Figure S3, crystal data see Table 1. ^1^H-NMR and ^13^C-NMR spectra were in line with published data (Table S3) [16,19,25].

Other phytochemical constituents from *C. indica* root extract and fractions were identified by gas chromatography–mass spectrometry (GC-MS). The system equipped with a 30 m × 0.25 mm I.D. × 0.25 μm film thickness DB-5MS column (Agilent Technologies, J & W Scientific Products, Folsom, CA, USA). The operating condition was imposed following the previous method [37]. In brief, 1 µL of the sample was injected into the GC system by an auto-sampler. The temperatures of injection port and detector temperature were set at 300 °C and 320 °C, respectively. The carrier gas was helium at inlet control with a split ratio of 5:1. The initial temperature of GC oven was 50 °C, after a boosting up to 300 °C with a rate of 10 °C /min, the oven temperature was maintained for 20 min. The mass range was recorded from 29 to 800 amu. The software JEOL’s GC–MS Mass Center System version 2.65a was occupied to analyze mass spectra of the natural components.

### 3.6. Quantification of Dentatin, Nordentatin and Clausine K contents by HPLC-UV/VIS

The contents of isolated compounds in *C. indica* root were quantified by the HPLC with a UV/Vis detector at 270 nm. The stationary phase was XBridge BEH Shield RP18 (130Å, 5 µm, 2.1 mm × 100 mm) column (Waters Cooperation, Milford, MA, USA). The mobile phase consisted of solution A (0.1% aqueous formic acid) and solution B (100% acetonitrile), which was adjusted in a gradient program as follows: 5% B during 0-2 min, a linear increase from 5 to 70% B during 2–12 min, 100% B from 12–16 min and maintained for 6 min, 100% B to 5% during 22–24 min and other 10 min for equilibration. The injection volume was 3 µL and the flow rate was 400 µL/min. The operation was carried out in 35 min under room temperature. The corresponding peaks from a sample were detected and their areas were used to calculate the amount (mg per g dry weight, mg/g DW) over the standard curve built from the isolated dentatin, nordentatin, and clausine K. The linearity of system was observed in the range of 5–100 µg/mL with correlation coefficients (r^2^) of 0.9992 for dentatin, 0.9991 for nordentatin, and 0.9996 for clausine K. The limits of detection (LOD) were 0.12, 0.13, and 0.09 ng/mL for dentatin, nordentatin, and clausine K, respectively, meanwhile, the limits of quantitation (LOQ) for these compounds were 0.36, 0.38, and 0.26 ng/mL.

### 3.7. Statistical Analyses

Results were displayed as mean ± standard error (SE). The statistically significant differences among samples were determined by ANOVA followed by Tukey’s multiple comparison test (*p* < 0.05). All observations were conducted three times (*n* = 3), and the data were analyzed by Minitab software version 16.0 (Minitab Inc., State College, PA, USA). The IC_50_ value was calculated by plotting the graph (Microsoft Excel, version 1902, Microsoft, Redmond, WA, USA) between the different concentrations of a tested sample and the corresponding percent inhibitions.

## 4. Conclusions

The bio-guided isolation of the ethyl acetate extract from *Clausena indica* roots achieved three known compounds dentatin, nordentatin, and clausine K. The compounds have been previously reported in *C. excavata*, but their presence in *C. indica* are described for the first time herein. Except for dentatin and fraction **Re1**, all fractions, nordentatin and clausine K exerted promising antioxidant activities. The fraction **Re2** is responsible for the anti-α-amylase effect while clausine K and the fraction **Re4** are pioneering tyrosinase inhibitors of *C. indica* roots. Bioactive constituents of the plant extracts and fractions were provisionally characterized by GC-MS, the most abundant analytes might belong to carbazole alkaloids. The modern techniques and further isolations should be implemented in exploiting and discovering prospective active compounds from this plant.

## Figures and Tables

**Table 1:** Crystal data for dentatin, nordentatin and clausine K.

	Dentatin		Nordentatin		Clausine K	
Chemical formula	C_20_H_22_O_4_		C_19_H_20_O_4_		C_15_H_13_NO_4_	
Molecular weight	326.39 g/mol		313.36 g/mol		271.26 g/mol	
Temperature	173(2) K		173(2) K		173(2) K	
Wavelength	0.71073 Å		0.71073 Å		0.71073 Å	
Crystal size	0.020 × 0.170 × 0.200 mm		0.080 × 0.130 × 0.250 mm		0.090 × 0.090 × 0.130 mm	
Crystal habit	clear pale-yellow plate		clear light-yellow block		clear yellow block	
Crystal system	triclinic		monoclinic		monoclinic	
Space group	P -1		P 1 21/n 1		P 1 21/n 1	
Unit cell dimensions	a = 9.330(8) Å	α = 102.188(9)°	a = 11.685(10) Å	α = 90°	a = 7.032(4) Å	α = 90°
	b = 9.472(8) Å	β = 105.805(9)°	b = 10.832(9) Å	β = 94.009(9)°	b = 14.522(9) Å	β = 99.276(7)°
	c = 10.380(9) Å	γ = 90.679(9)°	c = 12.596(10) Å	γ = 90°	c = 12.660(8) Å	γ = 90°
Volume	860.4(12) Å^3^		1590.(2) Å^3^		1275.9(13) Å^3^	
Z	2		4		4	
Density (calculated)	1.260 g/cm^3^		1.309 g/cm^3^		1.412 g/cm^3^	
Absorption coefficient	0.087 mm^−1^		0.091 mm^−1^		0.104 mm^−1^	
F(000)	348		668		568	

**Table 2:** Major phytochemical components of *Clausena indica* extracts analyzed by gas chromatography-mass spectrometry (GC-MS).

No.	Identified Compounds	RT (min)	MW (g/mol)	Peak Area in Extracts (%)						
				MRT	MRE	Re1	Re2	Re3	Re4	Re5
1	Palmitic acid	17.05	256	-	-	<	13.04	-	-	-
2	Seselin	18.27	228	7.0	8.5	12.4	2.4	-	-	2.9
3	l-5-propylthiomethylhydantoin	19.49	188	1.3	1.2	-	-	-	-	1.2
4	Braylin	19.55	258	9.9	11.4	13.7	6.1	-	-	-
5	**UC1**	20.24	230	<	<	-	-	58.7	26.1	-
6	Anisocoumarin B	20.36	246	-	-	-	-	-	14.1	-
7	**UC2**	20.85	244	-	<	-	<	2.1	<	2.3
8	Nordentatin	20.94	312	5.1	5.7	-	<	10.9	2.1	2.9
9	**UC3**	21.08	297	<	<	-	-	2.8	-	-
10	Clausine K	21.25	271	-	<	-	-	-	-	15.0
11	Dentatin	21.48	326	70.6	68.0	61.7	10.7	-	-	-
12	**CA1**	21.85	227	-	<	-	-	-	2.8	-
13	**CA2**	23.29	293	1.9	2.3	3.5	-	-	-	-
14	**CA3**	23.34	241	-	-	-	10.0	-	-	-
15	**CA4**	24.85	279	-	-	-	1.9	1.1	11.2	-
16	**CA5**	24.92	285	-	<	-	-	5.7	-	-
17	7-Hydroxyheptaphylline	25.80	295	-	-	-	-	-	9.2	-
18	Clionasterol	28.41	414	-	-	-	10.2	-	-	-

RT, retention time; MW, molecular weight; UC, unknown compound; CA, carbazole alkaloid; -, not detected; <, trace of peak area that was lower than 1%.

**Table 3:** Antioxidant activities of MR extracts, fractions, and isolated compounds.

Sample	DPPH Assay IC_50_ (µg/mL)	ABTS Assay IC_50_ (µg/mL)	β-Carotene Assay LPI (%)
MRT	273.8 ± 9.6 ^e^	277.5 ± 3.1 ^f^	73.5 ± 1.4 ^bc^
MRE	172.4 ± 1.2 ^d^	156.0 ± 1.3 ^d^	82.8 ± 1.1 ^a^
Dentatin	*ne*	*ne*	37.9 ± 0.8 ^f^
Nordentatin	49.2 ± 0.5 ^b^	69.9 ± 1.1 ^b^	69.8 ± 0.4 ^cd^
Clausine K	2197.8 ± 53.3 ^f^	5264.0 ± 164.0 ^g^	60.6 ± 1.1 ^e^
**Re1**	*ne*	*ne*	67.7 ± 0.4 ^d^
**Re2**	283.5 ± 7.0 ^e^	188.5 ± 3.6 ^e^	73.7 ± 0.1 ^bc^
**Re3**	88.1 ± 3.2 ^c^	149.4 ± 3.2 ^d^	76.4 ± 0.7 ^b^
**Re4**	32.4 ± 0.3 ^ab^	38.5 ± 0.8 ^a^	76.1 ± 0.5 ^b^
**Re5**	90.9 ± 2.5 ^c^	151.8 ± 2.4 ^d^	81.2 ± 0.8 ^a^
BHT	16.0 ± 0.2 ^a^	82.8 ± 1.1 ^c^	82.7 ± 0.3 ^a^

Data were mean ± standard error (SE) (*n* = 3); Similar superscript letters in a same column show nonsignificant differences among samples at *p* < 0.05. BHT, butylated hydroxytoluene; *ne*, negligible effect (inhibition < 10% at a maximum concentration of 10 mg/mL).

**Table 4:** α-Amylase, elastase and tyrosinase inhibitions of *Clausena indica* extracts, fractions and isolated compounds.

Sample	α-Amylase Inhibition IC_50_ (µg/mL)	Elastase Inhibition at 10 mg/mL (%)	Tyrosinase Inhibition IC_50_ (µg/mL)
MRT	1383.3 ± 14.3 ^d^	-	-
MRE	1004.6 ± 8.9 ^c^	-	-
Dentatin	-	-	-
Nordentatin	-	41.9 ± 0.8 ^a^	-
Clausine K	-	21.9 ± 0.8 ^b^	179.5 ± 1.7 ^a^
**Re1**	-	-	-
**Re2**	573.8 ± 14.3 ^b^	-	*ne*
**Re3**	-	-	1798.0 ± 20.3 ^c^
**Re4**	-	-	243.8 ± 2.7 ^a^
**Re5**	-	-	2897.3 ± 25.0 ^d^
**Control**			
Acarbose	39.6 ± 1.4 ^a^	*nd*	*nd*
Oleanolic acid (IC_50_)	*nd*	0.3 ± 0.0	*nd*
Myricetin	*nd*	*nd*	735.6 ± 7.2 ^b^

Data were mean ± standard error (SE) (*n* = 3); Similar superscript letters in a same column show nonsignificant differences among samples at *p* < 0.05; -, inactive (no inhibitory activity at a maximum concentration of 10 mg/mL); *ne*, negligible effect (inhibition < 10% at a maximum concentration of 10 mg/mL); *nd*, not determined.

## Supplementary Materials

The following are available online, Figures S1–S3: Total ion chromatography (**upper**) and tandem mass spectrometry (MS-MS) spectrum (**lower**) of dentatin, nordentatin, clausine K; Figure S4: GC-MS fragmentation patterns of **UC1** (**a**), **UC2** (**b**), and **UC3** (**c**) detected in *Clausena indica* roots; Figure S5: Dose-response curves for biological activities of major active constituents isolated from *Clausena indica* roots and positive controls; Figure S6: Linear curves used to calculate IC_50_ of biological activities of major active constituents isolated from *Clausena indica* roots; Table S1, S2, S3: ^1^H (400 MHz) and ^13^C (101 MHz) NMR spectral data of dentatin, nordentatin, clausine K.
