# Supplementary material for: Bio-Guided Isolation of Prospective Bioactive Constituents from Roots of Clausena indica (Dalzell) Oliv
Source: Molecules. 2019 Dec 4;24(24):4442. doi: 10.3390/molecules24244442 (PMC6943736; doi:10.3390/molecules24244442)
Supplement: Supplementary file 1 [file molecules-24-04442-s001.zip › Supplementary data_Figure S1-S4.pdf]

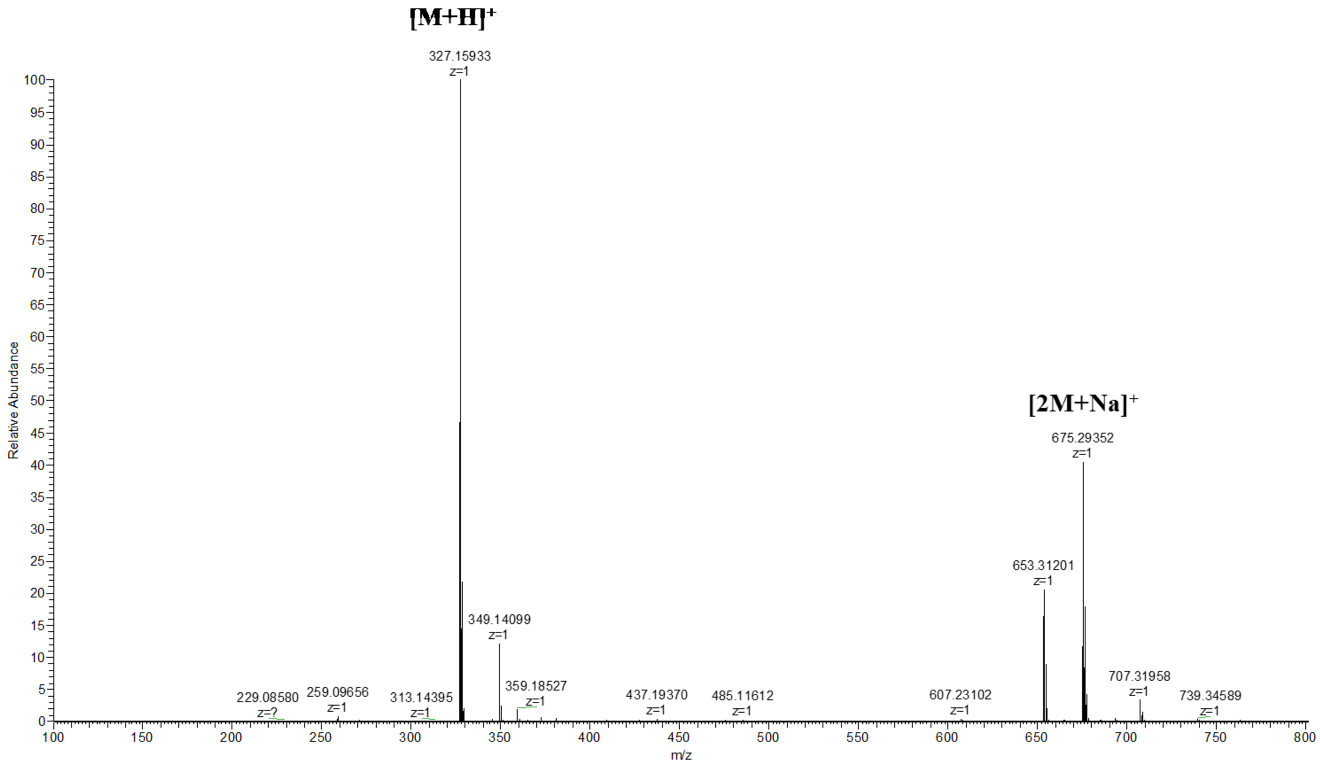

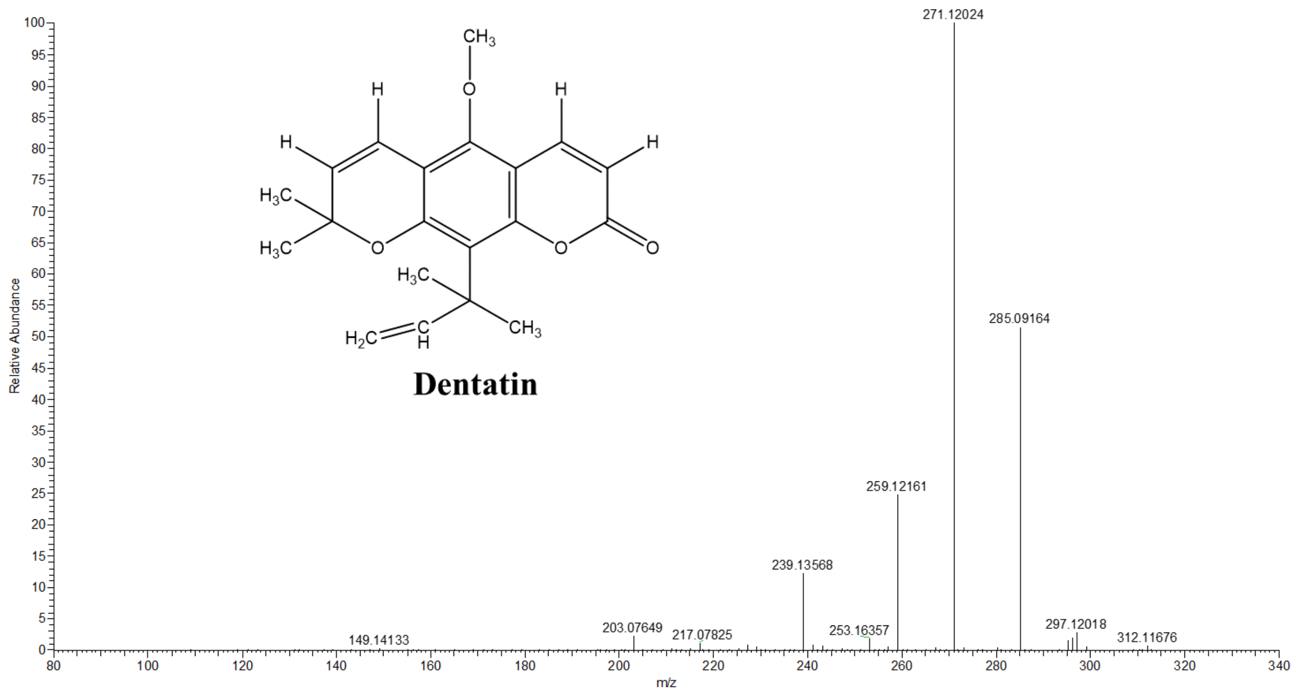

Figure S1. Total ion chromatography (upper) and tandem mass spectrometry (MS-MS) spectrum (lower) of dentatin by a positive model

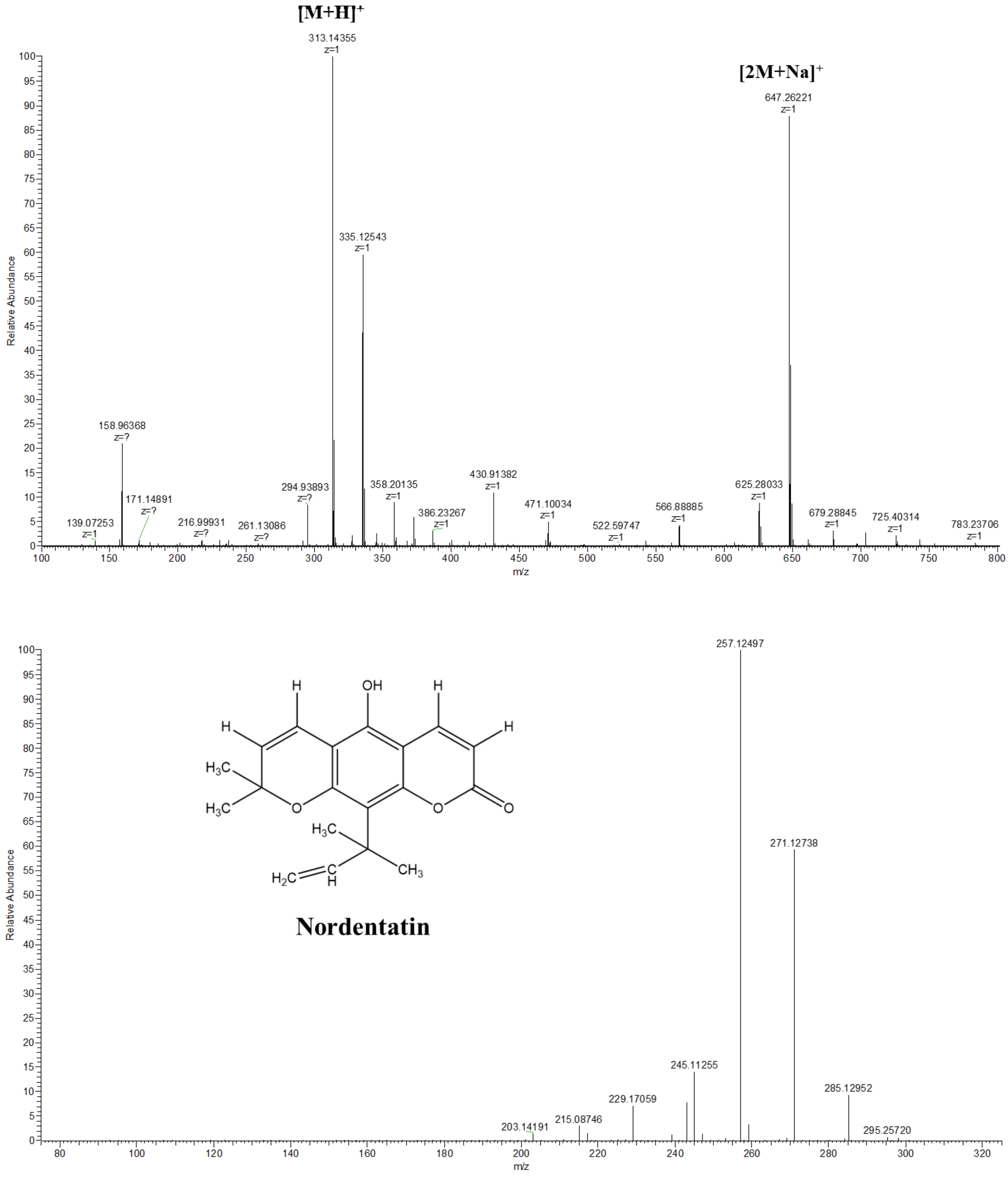

Figure S2. Total ion chromatography (upper) and tandem mass spectrometry (MS-MS) spectrum (lower) of nordentatin by a positive model

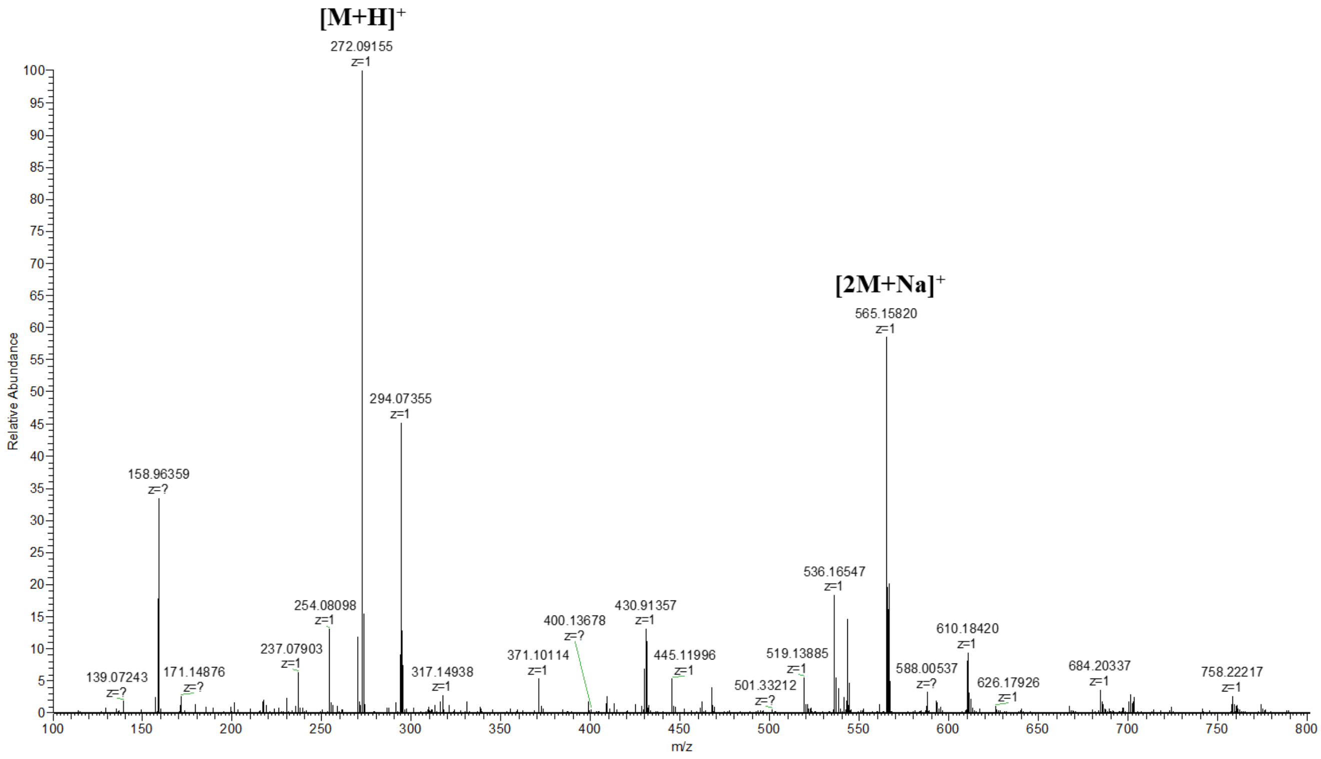

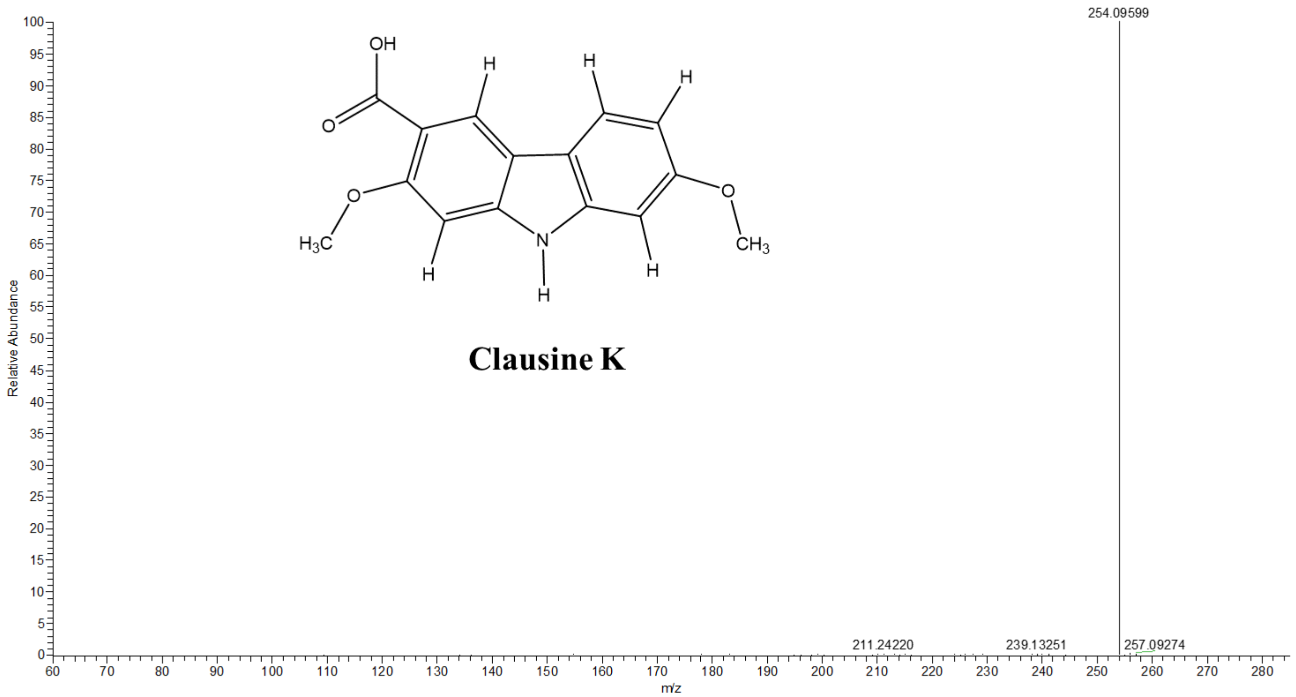

Figure S3. Total ion chromatography (upper) and tandem mass spectrometry (MS-MS) spectrum (lower) of clausine K by a positive model

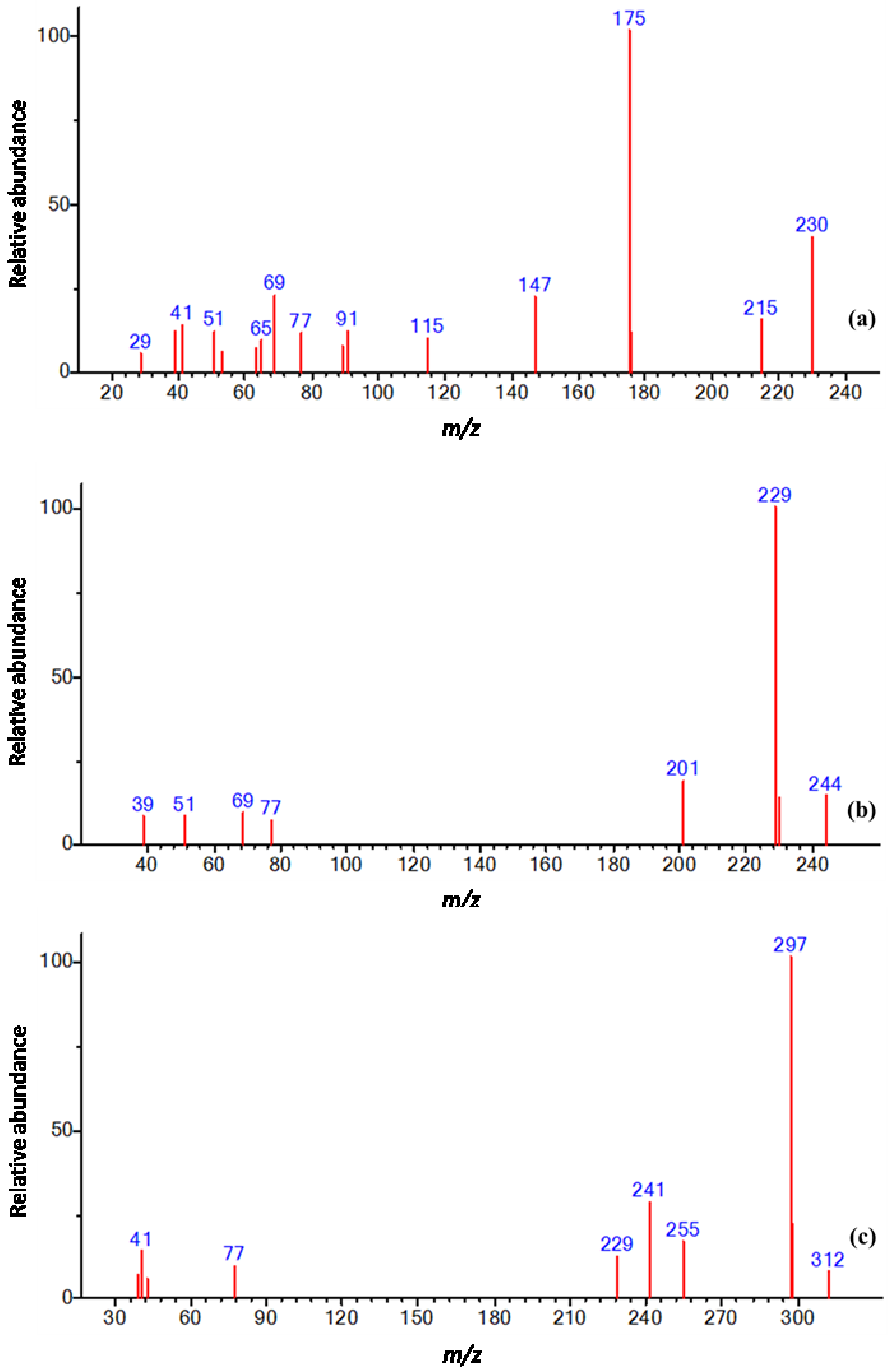

Figure S4. GC-MS fragmentation patterns of **UC1** (a), **UC2** (b), and **UC3** (c) detected in *Clausena indica* roots
