# Supplementary material for: Bio-Guided Isolation of Prospective Bioactive Constituents from Roots of Clausena indica (Dalzell) Oliv
Source: Molecules. 2019 Dec 4;24(24):4442. doi: 10.3390/molecules24244442 (PMC6943736; doi:10.3390/molecules24244442)
Supplement: Supplementary file 1 [file molecules-24-04442-s001.zip › Supplementary data_Figure S5&S6.pdf]

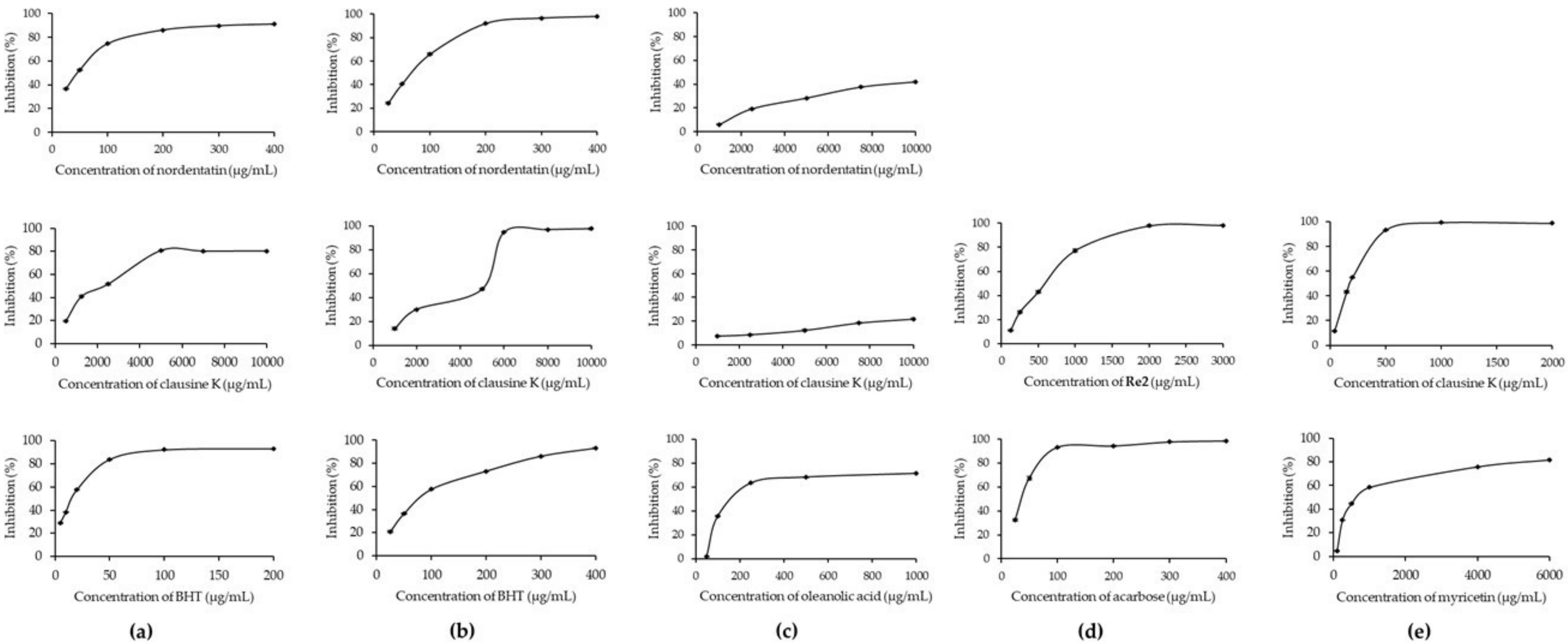

Figure S5. Dose response curves for biological activities of major active constituents isolated from *Clausena indica* roots and positive controls. The results are presented as mean ± standard error (n=3). BHT, butylated hydroxytoluene. **(a)** DPPH radical scavenging activity; **(b)** ABTS radical scavenging activity; **(c)** elastase inhibitory activity; **(d)** α-amylase inhibitory activity; **(e)** tyrosinase inhibitory activity.

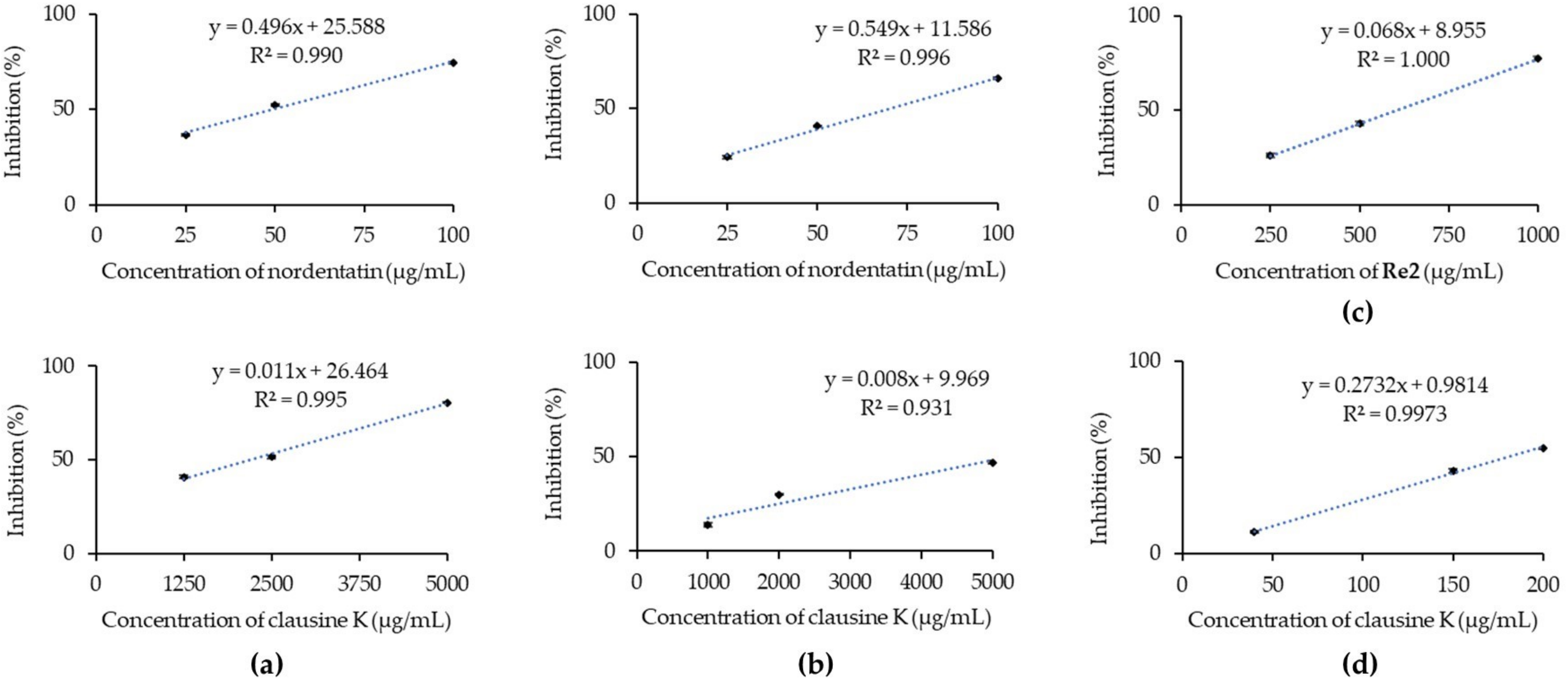

Figure S6. Linear curves used to calculate IC_{50} of biological activities of major active constituents isolated from *Clausena indica* roots. The results are presented as mean ± standard error (n=3). **(a)** DPPH assay for nordentatin (upper) and clausine K (lower); **(b)** ABTS assay for nordentatin (upper) and clausine K (lower); **(c)** α-amylase assay for fraction **Re2**; **(d)** tyrosinase assay for clausine K.
