# Supplementary material for: Bio-Guided Isolation of Prospective Bioactive Constituents from Roots of Clausena indica (Dalzell) Oliv
Source: Molecules. 2019 Dec 4;24(24):4442. doi: 10.3390/molecules24244442 (PMC6943736; doi:10.3390/molecules24244442)
Supplement: Supplementary file 1 [file molecules-24-04442-s001.zip › Supplementary data_Table S1_S3.pdf]

Table S1. ^{1}H (400 MHz, $CDCl_3$) and ^{13}C (101 MHz, $CDCl_3$) NMR spectral data of dentatin

Position	δ_H (mult., *J* in Hz)	δ_C
1a		154.01
2		160.75
3	6.19 (1H, d, 9.6)	111.71
4	7.87 (1H, d, 9.7)	138.92
4a		107.55
5		151.27
5a		108.3
6	6.57 (1H, d, 9.9)	116.36
7	5.70 (1H, d, 10.0)	130.40
8		77.39
9a		156.04
10		119.24
1′		41.20
2′	6.31 (1H, dd, 17.4, 10.6)	149.86
3′		108.21
3′a	4.88 (1H, d, 10.5)	
3′b	4.94 (1H, d, 17.4)	
4′ and 5′	1.69 (6H, s)	29.44
OCH_3	3.83 (3H, s)	63.43
$2CH_3 – 8$	1.45 (6H, s)	27.57

Table S2. ^{1}H (400 MHz, $CDCl_3$) and ^{13}C (101 MHz, $CDCl_3$) NMR spectral data of nordentatin

Position	δ_H (mult., *J* in Hz)	δ_C
1a		154.19
2		161.42
3	6.17 (1H, d, 9.6)	110.34
4	8.05 (1H, d, 9.6)	139.14
4a		103.86
5		146.53
5a		106.03
6	6.53 (1H, d, 9.9)	114.90
7	5.72 (1H, d, 9.9)	130.04
8		77.10
9a		155.91
10		116.23
1′		41.03
2′	6.30 (1H, dd, 17.4, 10.5)	150.06
3′		108.07
3′a	4.88 (1H, d, 10.6)	
3′b	4.94 (1H, d, 17.4)	
4′ and 5′	1.64 (6H, s)	29.56
OH	5.93 (1H, s)	
$2CH_3$-8	1.46 (6H, s)	27.30

Table Table S3. ^{1}H (400 MHz, DMSO) and ^{13}C (101 MHz, DMSO) NMR spectral data of clausine-K

Position	δ_H (mult., *J* in Hz)	δ_C
1	7.03 (1H, s)	94.39
1a		143.87
2		157.81
3		112.73
4	8.39 (1H, s)	123.60
4a		116.22
5	7.95 (1H, d, 8.5)	120.94
5a		116.76
6	6.78 (1H, dd, 8.5, 2.3)	108.58
7		158.57
8	6.98 (1H, d, 2.2)	95.51
8a		142.06
NH	11.28 (1H, br. s)	
COOH	12.11 (1H, s)	167.91
OCH_3-2	3.89 (3H, s)	56.41
OCH_3-7	3.83 (3H, s)	55.76
